# Research on automatic labeling of imbalanced texts of customer complaints based on text enhancement and layer-by-layer semantic matching

**DOI:** 10.1038/s41598-021-91189-0

**Published:** 2021-06-04

**Authors:** Xiaobo Tang, Hao Mou, Jiangnan Liu, Xin Du

**Affiliations:** 1 Center for Information System Research, Wuhan University, Wuhan, China; 2 School of Information Management, Wuhan University, Wuhan, China; 3 Sichuan Xichang Electric Power Co., Ltd., Sichuan, China

**Keywords:** Computer science, Information technology

## Abstract

Due to its potential impact on business efficiency, automated customer complaint labeling and classification are of great importance for management decision making and business applications. The majority of the current research on automated labeling uses large and well-balanced datasets. However, customer complaint labels are hierarchical in structure, with many labels at the lowest hierarchy level. Relying on lower-level labels leads to small and imbalanced samples, thus rendering the current automatic labeling practices inapplicable to customer complaints. This article proposes an automatic labeling model incorporating the BERT and word2vec methods. The model is validated on electric utility customer complaint data. Within the model, the BERT method serves to obtain shallow text tags. Furthermore, text enhancement is used to mitigate the problem of imbalanced samples that emerge when the number of labels is large. Finally, the word2vec model is utilized for deep text analysis. Experiments demonstrate the proposed model's efficiency in automating customer complaint labeling. Consequently, the proposed model supports enterprises in improving their service quality while simultaneously reducing labor costs.

## Introduction

As quality of life improves, customers have higher expectations for products purchased and services received. Major enterprises strive to remain aligned with customer interests by creating customer complaint channels that resolve customer disputes and dissatisfaction. In addition, with an increase in business competition, customer churn reduction becomes increasingly important. The critical aspect of reducing customer churn is customer satisfaction improvement. Thus, customer complaints, which reflect customer satisfaction, form an essential bridge between customers and companies.

Although the number of complaints is often large, enterprises commonly rely on manual methods for processing complicated complaint content. As a result, customer complaint processing requires significant labor and time. Since text mining enables automatic analysis of customer complaints, it can reduce labor costs, promote customer satisfaction, and prevent customer churn. Thus, research on text mining of customer complaints can play a crucial role in improving enterprises' efficiency.

**Figure 1:**
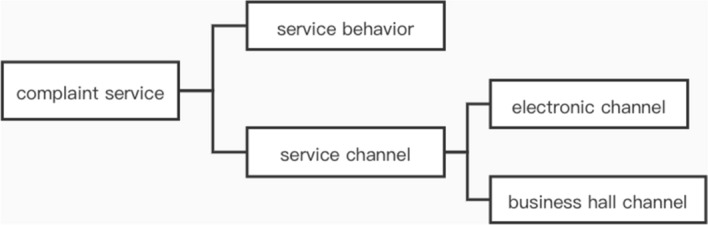
Example of the hierarchy of a customer complaint.

Automatic labeling and classification refer to the use of computational procedures that generate tags to summarize, describe, and classify textual content. Current research on automatic labeling of both short and long texts mainly relies on keyword extraction and topic modeling. However, the derived labels do not have a hierarchical structure. Customer complaint texts, on the other hand, are processed in accordance with the complaint classification, where the responsible customer service department is contacted, customer complaints are processed, and the processing results are returned. Based on the level of admissibility, complaint texts are usually divided into different business categories that can be visualized with a tree-like structure. For example, Fig. 1 shows that "complaint service" may be divided into "service behavior" and "service channel" on the first hierarchy level. The "service channel" may be further divided into an "electronic channel" and a "business hall channel" to form a second hierarchy level.

Therefore, the customer complaint labels are characterized by a hierarchical structure but also the excessive use of deep labels and a relatively small, imbalanced sample size. When a traditional autolabeling algorithm assigns a text to the lowest admissible business category, the text can be understood to automatically belong to the relevant higher admissible business category. However, such an approach does not make effective use of hierarchical information between categories. Further, customer complaints can have numerous deep labels, resulting in small and imbalanced samples. Such samples pose an obstacle in training an autolabeling model.

This paper addresses the described issues by proposing a BERT and word2vec-based automated customer complaint labeling model. The bidirectional encoder representations from transformers (BERT) method is a pretrained language representation model and is viewed as landmark work in the field of natural language processing (NLP). BERT makes full use of a large number of unsupervised texts for self-supervised learning and encodes linguistic knowledge. Experiments demonstrate its superior performance on various NLP tasks^1^. Word2vec is a technique used to calculate word vectors^2^. Based on a given corpus and a training model, word2vec quickly and efficiently represents a word in the form of a vector that enables the calculation of the word-to-word similarity.

The model presented in this paper enables the hierarchical classification of customer complaints. Within this work, class labels at the second hierarchical level (i.e., Level 2 of admissible business classes) are called shallow labels. In contrast, class labels for Level 3 admissible business classes are called deep labels. The model's automatic labeling of customer complaints is divided into two stages. In the first stage, the BERT classification algorithm is used to identify the shallow labels. In the second stage, the deep labels are determined by calculating the similarity between the text and labels to be matched. The class label with the highest similarity to the text is selected as the complaint label. The BERT-based classification method is used for shallow text labels, and a similarity calculation model based on word2vec is used for deep text labels. The contributions of this paper are as follows:

1. An automatic customer complaint text indexing model based on BERT and word2vec is proposed.
2. A text enhancement method is proposed to improve the problem of imbalanced text and improve the accuracy of the automatic indexing model.
3. In the process of matching the text with the indexing label, layer-by-layer semantic matching is adopted. First, the shallow label belonging to the text is determined, and then the deep label under the shallow label is used for text matching, rather than directly matching the text with the deep label, which significantly improves the accuracy of the automatic indexing model.

## Literature review

### Autolabeling and autoindexing

As noted in “Introduction”, autolabeling or autoindexing refers to the process of automatically assigning labels or tags to text to describe its content^3^. More precisely, automatic labeling is the process of extracting words and phrases directly from the original text to describe the document's subject matter.

Automatic indexing research began in 1957 when Luhn^4^ introduced computer technology into bibliographic studies and devised a word-frequency method based on Zipf's law. Earl^5^ combined syntactic analysis and statistical word-frequency methods to extract keywords. The work of Salton et al.^6^ applied the vector space model for autolabeling, whereas Deerwester et al.^7^ suggested the use of latent semantic analysis. Finally, Anjewierden and Kabel^8^ proposed an ontology-based method for automatic labeling. Hugo^9^ proposed an innovative method to address the complexity of events in medical event logs. Based on automatic labeling, similar events are clustered in potential space to create accurate labels. Su^10^ proposed an automatic evaluation and labeling architecture for product perceptual attributes based on a convolutional neural network.

### Imbalanced text

Imbalanced text is characterized by a significantly smaller number of elements in one category than in other categories. An important problem facing natural language processing and machine learning at this stage remains the efficient processing of imbalanced text in classification tasks. Major NLP tasks, including sentiment analysis, propaganda detection and event extraction from social media, are all examples of imbalanced classification problems.

Adinarayana^11^ proposed a hybrid imbalanced data learning framework (HIDLF) to address the imbalance of views in a movie review dataset and then classified the movie reviews by the proposed HIDLT-SVM algorithm. Harish^12^ proposed using a BERT model to address the problem of data imbalance in text classification. Li^13^ proposed a solution to the imbalanced text problem in a multiclassification task. The multiclass dataset is first decomposed into several binary datasets. Then, spectral clustering is used to divide a minority of the subset of binary categories into subspaces and oversamples them according to the characteristics of the data. Spectral clustering-based sampling takes into account the distribution of the data and effectively avoids oversampling outliers. After the data have reached an approximate equilibrium point, a multiclass classifier can be trained from these rebalanced data.

## BERT and word2vec-based model for automatic labeling of customer complaint

**Figure 2:**
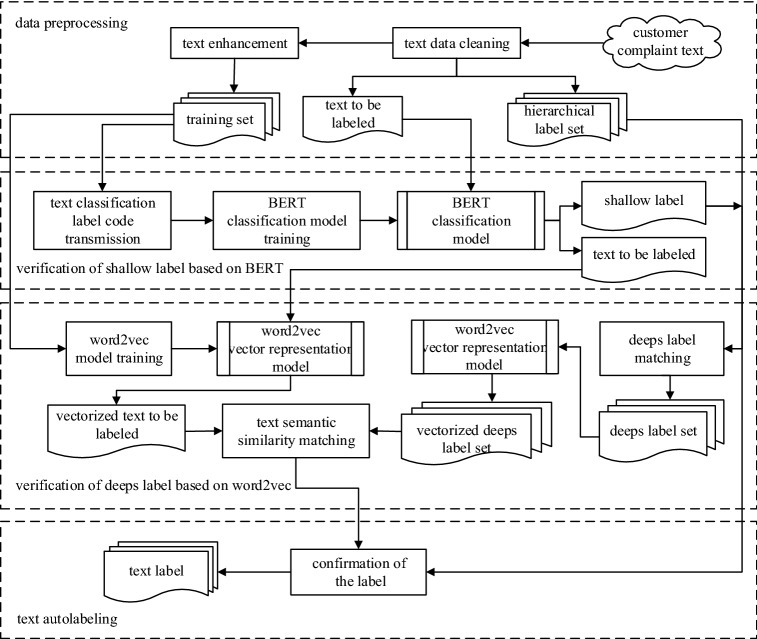
BERT and word2vec-based automatic text labeling model for customer complaints.

Figure 2 shows the proposed model for automatic customer complaint labeling based on BERT and word2vec. The model consists of four automatic text classification stages: data preprocessing, BERT-based text classification, word2vec-based semantic similarity matching, and label confirmation. BERT-based text classification is used to validate the shallow labeling scheme, and word2vec-based semantic similarity matching implements deep labeling. The model first confirms the shallow labels of the text to be indexed by using the BERT classification model. Then, according to the hierarchical label set, the deep label under the shallow label is found. Finally, the word2vec semantic similarity matching model is used to match the deep labels of the text to be indexed.

### Data preprocessing

Data preprocessing is a series of normative operations performed on the acquired irregular and chaotic texts. The goal of preprocessing is to reduce irregularities, remove inaccuracies, and reduce noise. This step serves as a basis for the subsequent text manipulation. Within this work, data preprocessing consists of the following steps:

1. Data acquisition: Through consultation and cooperation with the power company, customer complaint data are obtained, which includes the customer complaint text and the corresponding tag.
  Among them, the label is divided into a first-level label, a second-level label and a third-level label. For example, a customer submitted the following complaint: “The customer asked a staff member to deal with the power failure at home, but the staff member did not deal with it and did not explain the reason to the customer.” The text belongs to the "service attitude of other personnel" (third-level label) under "service behavior" (secondary label) under "service complaint" (first-level label).
2. Data cleaning: Data cleaning mainly includes text filtering and hierarchical label set extraction.
  Text filtering mainly manually filters the customer complaint text. Due to the freedom of customer complaints, there are some meaningless noisy data, so it is necessary to rely on manual screening of the obtained data.
  The extraction of the hierarchical label set organizes the indexing labels of the text data to form a hierarchical label set.
3. Text enhancement: The (too) deep labels lead to a few samples in several categories. Thus, the classification process commonly deals with imbalanced samples that affect classification accuracy. Within this work, this problem is addressed by enhancing the texts falling in critical categories.

Oversampling is the process of generating a bias towards selecting the data from specific categories. In other words, it is a data enhancement technique that increases the likelihood of choosing a positive case, thus overcoming the issues stemming from the imbalance of positive and negative samples. However, the adjusted dataset will often be much larger than the original dataset, increasing the time overhead.

The main methods of data enhancement in NLP are adding noise and paraphrasing. Noise addition modifies a positive case by, for example, randomly deleting certain words or disrupting the word order to generate new data. Paraphrasing can be seen as a seq2seq task. For example, in question-and-answer systems, question retelling is used to improve the question format. Then, the new question is used instead of the old question in the question-and-answer system.

Image pixels commonly contain noise, which often does not have a significant impact. However, in this study, a small change in a text may greatly impact the task since the deleted words are likely important. Removing an unimportant word, on the other hand, is equivalent to deleting a stop word. Disrupting the order of many word is even more infeasible, as sentence disruptions remove the contextual relationship among words. Thus, text generation is seen as a more effective way to enhance the texts within this work.

In this paper, data enhancement is divided into two steps. The first step extracts the texts from underrepresented categories and translates them. The texts are translated from Chinese to English and back. The newly obtained Chinese texts contain different expressions and can be saved as new texts under the category tag. This procedure doubles the number of complaints in the category. The second step—synonym substitution—has to be done to increase the number of texts even further.

The second step uses the Harbin Institute of Technology (HIT) thesaurus and the partitioning tool in the NLP package pyltp. First, the thesaurus is traversed, and a synonym list is constructed for each word in the sentence. For example, if a sentence contains the word "pay", the list of synonyms include "compensate, give, refund, repay, pay off…". One restriction is introduced. Namely, synonym replacement is not performed for very short words since they are commonly adverbs or prepositions (e.g., "of" or "in"), for which synonym replacement is not very meaningful. Next, the three top-ranking synonyms are selected as a final result for each word in the synonym list. If a word has less than three synonyms (e.g., "customer"), the exact word is copied three times (the synonym list becomes "customer, customer, customer"). In this manner, each word in the sentence is associated with three words, and a simultaneous replacement of multiple words in the sentence generates the new sentence. Since the thesaurus is not comprehensive and short words are not replaced, the original sentence parts are retained, while the other parts are replaced with synonyms. For example, the following three instances are obtained from a single sentence:

- The customer paid 400 RMB at the State Grid Pengshan Power Supply Office this morning.
- The customer refunded 400 RMB at the State Grid Pengshan Power Supply Office before noon.
- The customer repaid 400 RMB at the State Grid Pingshan Power Supply Office half a day before.

Following the described synonym substitution procedure, each original sentence can be expanded into three sentences. If additional data are required, more sentences can be generated by utilizing longer synonym lists. Substitution by synonyms introduces a level of randomness in the sentences, forcing the model to learn deeper levels of semantic information and, consequently, enhancing the model robustness and reducing the influencing data factors. Using the HIT thesaurus has at least two advantages. First, using the thesaurus eliminates the need to calculate similarity and thus avoids the inaccuracies in calculating the word2vec similarity caused by scarce customer complaints data. Second, the HIT thesaurus does not distinguish between specific domains. Several words in the electricity field may not have synonyms, such as "State Grid Pengshan Power Supply Business Hall". Using thesaurus-based synonym replacement can, to a certain extent, alleviate the emergence of fluency problems.

### Verification of shallow labels based on BERT

Text is unstructured data. To rely on a computer for text classification, the text must be transformed into computer-comprehensible structured data. Therefore, text representation is crucial for natural language processing tasks. Traditional word vector representation models include the TF-IDF model and word2vec neural network language model. The TF-IDF model performs vector representation of the text based on the product of the term frequency (TF) and inverse document frequency (IDF); word2vec maps words to a low-dimension and high-density vector space by training a neural network model. However, these text representation models have limitations: they do not consider the meaning of words in context. Taking the word “book” as an example, in the sentence “I am reading a good book on economics”, “book” means a written work or composition that has been published. In the sentence “the agent booked tickets to the show for the whole family”, “book” means arrange for and reserve (something for someone else) in advance. A traditional word vector used to represent the model cannot effectively combine context and distinguish semantics, which is obviously unreasonable. To solve this problem, this paper uses the BERT model for text representation.

In recent years, several studies have successfully mitigated the listed problems by pretraining deep neural network models and fine-tuning them to perform specific NLP tasks. BERT is one such pretrained language model with output vectors, a word-level vector, and a sentence-level vector. Sentence-level vectors can capture the entire sentence's semantics and are often used in classification tasks.

In this paper, a BERT-based text classification model is proposed. Every BERT sequence starts with a special classification token denoted by CLS. The model first takes the CLS vector representing the sentence, and then the sequence is passed to a fully connected layer. The model uses a binary cross-entropy loss function and the softmax function, which is an activation function typically used in multiclassification tasks.

**Figure 3:**
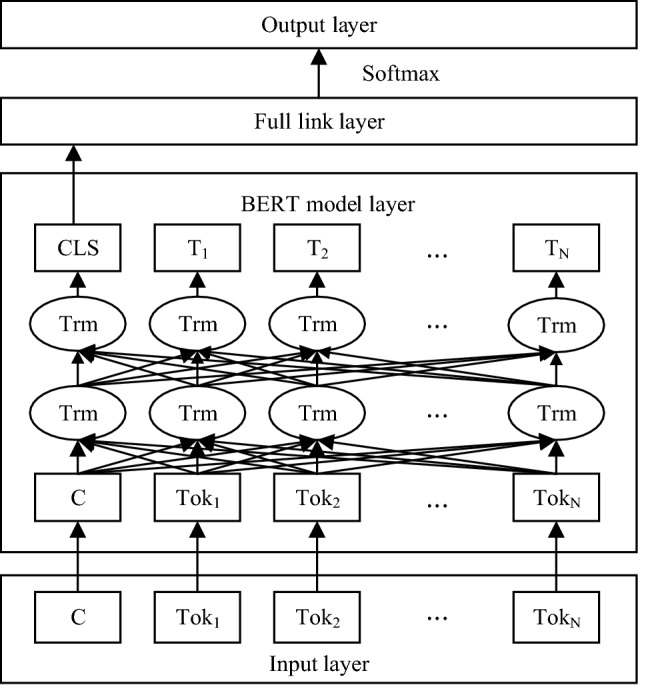
BERT-based text classification model.

The specific structure of the model is shown in Fig. 3. In the figure, ${Tok}_{1},{Tok}_{2},\dots ,{Tok}_{N}$ is the input vector at the word level, *C* is the input vector at the sentence level, ${T}_{1},{T}_{2},\dots ,{T}_{N}$ is the BERT model output vector at the word level, and CLS is the BERT model output vector at the sentence level.

To enable text classification using the model, the class labels must first be encoded and transformed. The class labels of each sample are mapped to a list $\left[{l}_{1},{l}_{2},\dots ,{l}_{N}\right]$, where *N* is the total number of class labels and ${l}_{i}\in \left\{\mathrm{0,1}\right\}, \forall i=1, 2, \dots N.$ When ${l}_{i} =1$, the sample obtains class label ${l}_{i}$.

Once the text is represented using BERT, the CLS vector is extracted and passed to the full link layer. The softmax function normalizes the output nodes' values so that the sum of the output nodes equals 1. The softmax function is defined as:

1 $$ soft\max Z_{j} = \frac{{e^{{z_{j} }} }}{{\mathop \sum \nolimits_{k = 1}^{K} e^{{z_{k} }} }} $$

After using text enhancement to solve the problem of category imbalance, this paper uses a BERT-based text classification model to classify customer complaint texts and uses the classification label as the text indexing label of the text at the shallow level.

The next step is to find the deep label under the shallow label based on the shallow label obtained by the BERT-based text classification model and the level-level label set obtained by data cleaning and use the text semantics based on word2vec similarity matching to determine the deep tags.

### Verification of deep labels based on word2vec

At present, there are many studies on question similarity matching, which can be roughly divided into question similarity research based on strings, question similarity research based on vector space models and question similarity research based on deep learning. The focus of this paper is not to propose a highly accurate or innovative question similarity matching model but to propose a set suitable for this problem and effective solutions, so this paper uses the commonly used question similarity calculation method: based on word2vec text vector representation, the cosine similarity of the calculation vector is the text semantic similarity.

**Figure 4:**
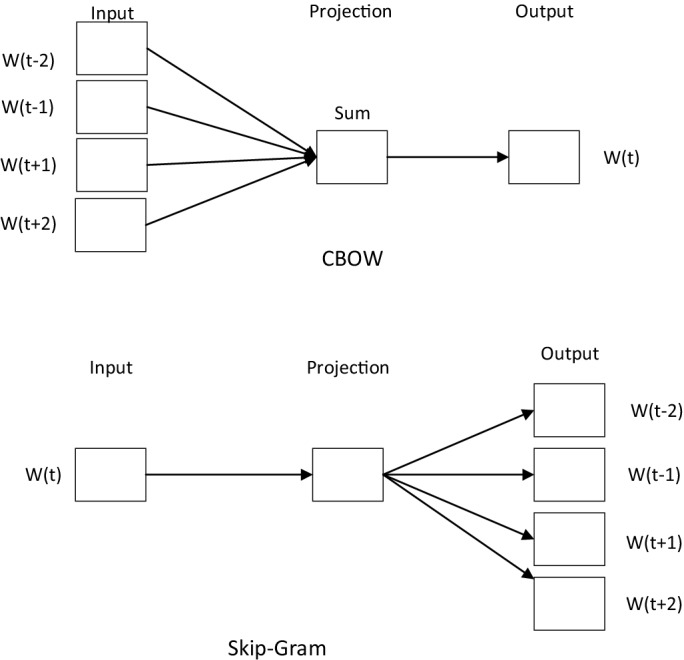
Two models of word2vec.

Word2vec uses two models, the continuous bag-of-words (CBOW) model and the continuous skip-gram model. The goal of the CBOW model is to predict the probability of a word based on the current context, while the skip-gram model does the opposite and determines the probability of a context based on the presented word (Fig. 4). Both models use artificial neural networks as their classification algorithms. Initially, each word is a random *n*-dimensional vector, but—upon training—the models obtain an optimal vector for each word.

Word2vec trains both CBOW and skip-gram models on a given corpus, and the output yields vector representations of all the words that appear in the corpus. The obtained word vectors enable the calculation of word-word relationships, such as word similarity and semantic relatedness.

This paper uses training set text data to train the word2vec text vector representation model. Through the shallow label obtained by the previous text classification model based on BERT and the hierarchical label set obtained by data cleaning, the deep label under the shallow label is found. Word2vec creates a vector representation of the index text and deep label. By calculating the cosine similarity between vectors, the deep label to be matched with the highest similarity is taken as the deep label of the text.

### Text autolabeling

The last phase of the autolabeling process deals with determining the final label from shallow and deep text labels. At the shallow level, the label is validated using the text-enhanced BERT classification model. Similarly, at the deep level, the validation process relies on word2vec to calculate the semantic similarity between the text and the potential labels. The final text label is determined by combining the shallow and deep citation labels.

## Experiments and analysis of results

### Data validation

A power company provided anonymized experimental data to protect the privacy of its customers. The experimental data consist of the electricity complaint text and the primary, secondary, and tertiary business categories to which the text belongs. The experiments reported in this paper were performed on the first- and second-level categories. The data were cleaned to ensure the accuracy of the experiment. After the initial manual screening and the removal of noisy data, a total of 16 shallow labels corresponding to 3438 complaints were selected as the experimental dataset. The distribution of the experimental dataset is shown in Table 1.

**Table 1:** Distribution of the samples in the secondary categories.

Serial number	Category tag	Number of texts containing the tag
1	Electricity construction	225
2	Electricity supply facilities	52
3	Voltage quality	557
4	Power supply reliability	1434
5	Power supply frequency	11
6	Acts of service	403
7	Service channels	29
8	Repair service	143
9	Power outage issues	58
10	Power outage information bulletin	51
11	Meter reading and reminders	86
12	Electricity tariffs	22
13	Power metering	138
14	Business expansion report	138
15	Operating charges	34
16	Change in electricity consumption	57

### Experimental procedures

In the data preprocessing stage, to improve the BERT classification effect, this paper uses the text enhancement method to expand samples with too little text under the indexing label. There are two main text enhancement methods used in this paper. The first method is to first translate the Chinese text into English and then translate the English text back to Chinese to obtain text with the same meaning but different expressions. The second method is to replace synonyms, using the word segmentation tool in HIT pyltp and a thesaurus to replace some words in a sentence with their synonyms to expand the corpus. After text enhancement, there are 3698 text data points, and the data distribution is shown in Table 2.

**Table 2:** Distribution of the complaints after text enhancement.

Serial number	Category tag	Number of texts containing the tag
1	Electricity construction	225
2	Electricity supply facilities	87
3	Voltage quality	557
4	Power supply reliability	1434
5	Power supply frequency	20
6	Acts of service	403
7	Service channels	47
8	Repair service	143
9	Power outage issues	101
10	Power outage information bulletin	86
11	Meter reading and reminders	152
12	Electricity tariffs	34
13	Power metering	138
14	Business expansion report	138
15	Operating charges	63
16	Change in electricity consumption	70

As described, the BERT-based text classification algorithm is first used to determine the shallow label for every complaint. The data were divided into training and test sets at a 9:1 ratio, yielding a test set of 369 complaints. Within the BERT classification model, the initial learning rate was set to 0.00001. Furthermore, the Adam optimizer was utilized, and the number of epochs was set to 2. For deep labels, the word2vec model was trained on an enhanced dataset with a dimensionality set to 100, and the resulting word2vec model was used for semantic similarity matching.

The model's performance was evaluated using the accuracy, precision, recall, and F1 score measures. The measures are defined below; TP denotes "true positives" (i.e., the number of texts correctly recognized as belonging to a class), and TN stands for "true negatives" (i.e., the number of texts correctly identified as not belonging to a category). FP and FN stand for "false positives" and "false negatives", respectively, i.e., the number of complaints falsely classified as either belonging or not belonging to a class.

1. The accuracy denotes the probability that the classifier correctly classifies a sample. In other words, it is the ratio of the number of correctly classified samples to the total number of samples:
  2 $$Accuracy= \frac{TP+TN}{TP+TN+FP+FN}$$
2. The precision is the ratio of true positives to the number of samples the classifier assigned to the class:
  3 $$P= \frac{TP}{TP+FP}$$
3. The recall is the ratio of true positives to the number of truly positive samples. In other words,
  4 $$R= \frac{TP}{TP+FN}$$
4. The F1 score is the harmonic mean of P and R and is calculated as follows:
  5 $$F1= \frac{2\times P\times R}{P+R}$$

### Analysis of the results

#### Analysis of the shallow autolabeling results

The shallow labeling results are shown in Table 3. The table reports the overall accuracy (i.e., the accuracy of all the samples and categories), average accuracy (i.e., the average categorical accuracy), average recall, and average F1 score. The results for each category are shown in Table 4.

**Table 3:** Overall evaluation of the shallow text citation tags.

Model	Overall accuracy	Average accuracy	Recall	F1 score
BERT text classification after text enhancement	0.9175	0.9279	0.9175	0.9168

**Table 4:** The results of the BERT model's shallow labeling by categories.

Serial number	Accuracy	Recall	F1 score
1	0.5000	0.6000	0.5455
2	0.9545	0.9545	0.9545
3	1.0000	0.9963	0.9982
4	0.6154	0.8889	0.7273
5	0.5000	0.5000	0.5000
6	0.8750	0.9333	0.9032
7	0.9286	0.8125	0/8667
8	0.8889	0.8000	0.8421
9	0.9130	0.6562	0.7636
10	1.0000	0.1667	0.2857
11	0.6962	0.8333	0.7586
12	0.4167	0.7143	0.5263
13	0.5000	0.7500	0.6000
14	0.9773	0.8776	0.9247
15	0.9664	1.0000	0.9829
16	1.0000	0.8462	0.9167

We also choose to use the LR classification models of BERT without text enhancement and TF-IDF with text enhancement to conduct experiments. The performance results are shown in Table 5.

**Table 5:** Model comparison results.

Model	Overall accuracy	Average accuracy	Recall	F1 score
Text-enhanced BERT model	0.9175	0.9279	0.9175	0.9168
Unenhanced BERT model	0.9073	0.8672	0.8642	0.8548
TF-IDF-LR model	0.8960	0.9011	0.8960	0.8899

The table shows that the text-enhanced BERT model outperforms both the BERT model (without enhancement) and the TF-IDF-LR model with respect to every measure. The TF-IDF-LR model had lower overall accuracy, recall, and F1 score than the BERT model applied to the nonenhanced dataset. The results for each category are shown in Table 6 (for the BERT model without text enhancement) and Table 7 (for the TF-IDF-LR model).

**Table 6:** The results of the BERT model's shallow labeling (without text enhancement) by category.

Serial number	Accuracy	Recall	F1 score
1	1.0000	0.2500	0.4000
2	0.8750	0.9545	0.9130
3	1.0000	0.9961	0.9980
4	0.5333	0.8889	0.6667
5	0.0000	0.0000	0.0000
6	0.7647	0.8667	0.8125
7	0.8824	0.9375	0.9091
8	1.0000	0.6842	0.8125
9	0.8889	0.5000	0.6400
10	0.5000	0.5000	0.5000
11	0.6667	0.8065	0.7299
12	0.5000	0.8571	0.6316
13	1.0000	0.5000	0.6667
14	1.0000	0.8776	0.9348
15	0.9800	0.9899	0.9849
16	0.8571	0.9231	0.8889

**Table 7:** The results of the TF-IDF-LR model's shallow labeling by category.

Serial number	Accuracy	Recall	F1 score
1	1.0000	0.5455	0.7059
2	0.8421	0.8000	0.8205
3	0.9890	1.0000	0.9945
4	1.0000	0.7692	0.8696
5	0.0000	0.0000	0.0000
6	0.8889	0.6667	0.7619
7	0.7143	0.7143	0.7143
8	0.8571	0.7059	0.7742
9	0.9167	0.6875	0.7857
10	0.5000	0.3333	0.4000
11	0.6047	0.9286	0.7324
12	1.0000	0.5333	0.6957
13	0.0000	0.0000	0.0000
14	0.9535	0.8367	0.8913
15	0.9821	0.9910	0.9865
16	0.9286	0.9286	0.9286

Table 6 shows that BERT achieves an accuracy of 0.0 for the fifth category. The training set for this category is very small, containing only two complaints. Similarly, TF-IDF-LR achieves accuracies of 0.0 for the fifth category and the 13th category, whose training set includes nine texts. However, the text-enhanced BERT model does not show the result of 0.0000, which improves the poor performance of the BERT model when there are too many index tags and too little text, which proves the necessity of text enhancement in this paper.

#### Analysis of the deep autolabeling results

The deep labeling results are shown in Table 8. The table shows that when the text is correctly classified on the shallow level and word2vec semantic similarity matching performed on the deep label, the model performs well in terms of the accuracy, precision, recall, and F1 score. The results are substantially improved compared to direct word2vec-based semantic similarity matching on the texts. These results demonstrate the proposed model's validity regarding performing classification before the similarity calculation.

**Table 8:** Comparison of the results of the semantic similarity calculation methods.

Methodology	Overall accuracy	Average accuracy	Recall	F1 score
Use layer-by-layer semantic matching first and then calculate similarity with word2vec	0.7357	0.7350	0.7357	0.7173
Calculate the similarity directly with word2vec	0.3018	0.4035	0.3018	0.3313

Using the method of semantic matching layer by layer and similarity calculation to automatically label the customer complaint text, the results are shown in Table 9.

**Table 9:** Autolabeling results (partial).

Serial number	Business level	Secondary operations type	Receiving business Type III	Textual content	Deep labels
1	Business complaints	Electricity tariffs	Tariff	After the meter was changed here, the staff indicated that the meter was in arrears and charged the…	Tariff
2	Business complaints	Electricity tariffs	Tariff	Unreasonable charge for electricity penalty (customer says monthly number. Even if you have paid the bill but do not renew the invoice…)	Tariff
3	Business complaints	power metering	Rotation, household meter conversion	Without the customer's knowledge… many times to the customer meter into…	Meter wiring error
…	…	…	…	…	…
664	Service Complaints	Acts of service	Service attitude of other personnel	After reflecting the farm network charges. Subsequently, the staff of the Zhongqiang power supply house telephone contact with the user…	Service attitude of the electrical inspectors
665	Service Complaints	Service Channels	E-channel Services	I am not going to be able to buy electricity, but I still cannot buy electricity	E-channel Services
666	Service Complaints	Acts of service	Service Standard for Business Office Personnel	In the process of transferring the name change, the customer asks if he can print out the previously unchanged VAT invoice…	Service attitude of the electrical inspectors

## Conclusion

This work tackles the problems emerging from the hierarchical structure of customer complaints, where too many deep labels result in small category sizes and imbalanced samples. This paper proposes a new model based on BERT and word2vec that enables the automatic labeling of customer complaints. This model uses text enhancement to mitigate the problem of small category sizes without changing the semantics. In accordance, the model improves the sample size balance.

The developed model relies on the BERT model to determine shallow labels and uses the word2vec model to derive deep labels, thus taking full advantage of the hierarchical characteristics of customer complaint labels.

The innovations of this paper are as follows. (1) An automatic indexing model of customer complaint text based on BERT and word2vec is proposed. (2) Before determining the shallow text indexing label based on BERT, we first process the text within a category with a very small sample size. By text enhancement, we can obtain more samples and retain the semantics of the samples to improve the problem of insufficient model training caused by the small sample size and class imbalance. (3) First, the shallow label is determined, and then the deep label under the shallow label is used for similarity matching, which makes full use of the hierarchical structure between labels and greatly improves the accuracy.

The experiments demonstrated the model's feasibility and validity. Nevertheless, there are several limitations to this work. The first limitation is the issue of data availability since, due to the unique nature of the customer complaint data, the data can be obtained only through cooperation with a power company. Furthermore, since the data are not publicly available, the data quality cannot be determined. Finally, the quality of the enhanced text can be improved by advancing the developed algorithm. Future work will be directed at optimizing the details and improving the model to advance autolabeling.

## Abbreviations

BERT: Bidirectional encoder representations from transformers
NLP: Natural language processing
CLS: An arbitrary symbol that can be replaced with something else
HIT: Harbin Institute of Technology
